# Range of abduction in patients with Legg-Calvé-Perthes disease – a nationwide register-based cohort study

**DOI:** 10.1186/s12891-020-03705-4

**Published:** 2020-11-05

**Authors:** Ahmad El-Harbiti, Yasmin D. Hailer

**Affiliations:** Section of Orthopaedics, Department of Surgical Sciences, Section of Paediatric Orthopaedic Surgery, Uppsala University, Uppsala, Sweden

## Abstract

**Background:**

Range of abduction often decreases during Legg-Calvé-Perthes Disease (LCPD) disease. However, a good range of abduction is required during the course of LCPD, especially when containment surgery should be performed. This study aimed to investigate how many patients registered in the Swedish Pediatric Orthopedic Quality register (SPOQ) with LCPD had reduced range of abduction at diagnosis in relation to sex or age at diagnosis or severity of disease (lateral pillar class at the time at diagnosis), if physiotherapy (PT) was prescribed and has a beneficial impact in maintaining (or increasing) abduction and if the range of abduction at diagnosis before fragmentation stage is predictive for the lateral pillar classification at fragmentation stage.

**Methods:**

The national Swedish Pediatric Orthopedic Quality Register (SPOQ), established in 2015, is used to identify patients with LCPD. The patients are registered at three time points: at diagnosis, at potential surgery and 2 years after diagnosis. Range of abduction and information on PT are required to register at all registration sessions. One hundred ninety-nine hips from 192 children were registered in the SPOQ.

**Results:**

Of all hips, the mean range of abduction at diagnosis was 39 degrees (range 0 to 90). One hundred twenty-six patients (63%) either received instructions for PT or were referred to a physiotherapist; two patients were treated additionally with an abduction brace. There was a trend that patients who received PT, compared to patients without PT, either maintained or increased their range of abduction at the 2-year follow-up. Older age at diagnosis correlated with decreased range of abduction at the 2-year follow-up (Estimate [Est]: − 3.1, 95% confidence interval [CI]: − 4.4 to − 1.7). The degree of abduction at diagnosis before fragmentation stage correlated with the lateral pillar group at the fragmentation stage (Est: -5.3, 95% CI: − 10.0 to − 1.1).

**Conclusion:**

In all, 63% of the children with LCPD in SPOQ received either written instructions or were referred to PT or both. PT seems to have a favorable impact for maintaining the range of abduction in children with LCPD. Children with a lower range of abduction at diagnosis (before the fragmentation stage) developed a higher degree of lateral pillar involvement as measured by the lateral pillar classification.

## Background

Early symptoms of Legg-Calvé-Perthes Disease (LCPD) can initially be discrete and the clinical presentation may vary. However, pain and a decrease in range of motion of the hip, especially in abduction and rotation, are relatively common [15]. Range of abduction at diagnosis and during the disease progression is crucial for the choice of treatment and outcome. The severity of LCPD is determined on radiographs according to the lateral pillar classification [4, 6], which is predictive for the prognosis and a tool for decision making as to whether surgery is needed. A drawback of the lateral pillar classification is that it measures the deformation of the femoral hip epiphysis’ lateral quarter, which appears first at the Waldenström’s fragmentation stage [7, 9]. A clinical method to determine the outcome already at early stages and that enables decision making for surgical treatment to prevent deformation would be eligible.

Treatment of LCPD aims to minimize the femoral head enlargement and asphericity and by that hinge abduction. The round acetabular socket is believed to mold the femoral head into a spherical shape when placed deep in the acetabulum in early stages [12, 13]. The goal is to place the lateral pillar of the femoral head under the acetabular roof to avoid the extensive enlargement of the femoral head. This concept has been the basis of both nonsurgical and surgical treatment options [1, 11, 13]. Nonsurgical treatment of Perthes’ disease mainly aims to maintain abduction either by physiotherapy (PT) or with orthoses or Petrie-casting. An alternative approach to nonsurgical treatment is to change the hip and its bony anatomy by performing Varus osteotomy of the proximal femur, Salter osteotomy or Triple osteotomy of the pelvis [8, 16, 17]. All these surgical techniques reduce the range of hip abduction temporarily. A good range of abduction is therefore required before performing surgery. Whether physiotherapy could help to maintain or even increase the range of abduction in children with LCPD is still unclear. Only a few studies address abduction or the effects of physiotherapy on the range of motion of the hip in children with LCPD [2, 13, 18].

The purpose of this study was (1) to investigate how many patients registered in the Swedish Pediatric Orthopedic Quality register (SPOQ) with LCPD had reduced range of abduction at the time of diagnosis in relation to sex or age at diagnosis or severity of disease (lateral pillar class at the time at diagnosis), (2) to investigate which patients received instructions or were referred to a physiotherapist to increase range of abduction (a) and if physiotherapy had any beneficial effects on maintaining or even increasing range of abduction by comparing the range of abduction at diagnosis and at a 2-year follow up (b) and (3) to investigate whether the range of abduction before the fragmentation stage is predictive for the severity of LCPD as assessed by lateral pillar classification.

## Patients and methods

To conduct this register-based cohort study we used data from the SPOQ, which gathered information on patients with LCPD nationwide. The SPOQ was established in 2015. All patients with a Swedish personal identification number, a radiographic confirmed LCPD, aged between 2 and 12 years at diagnosis and treated in Sweden since diagnosis should be registered in the SPOQ. Registration time points are time at diagnosis, time at primary surgery (and possible secondary surgeries), time at a 2-year follow-up after diagnosis, time at the age of 10 and 18 years. Registration variables are patient’s characteristics (e.g. date of birth, sex, length, weight), radiographic assessments (e.g. LP, articulo-trochanteric distance and Reimer’s index at 2-, 10- and 18 years follow-up) and clinical assessments (e.g. range of abduction, internal and external rotation, Trendelenburg’s sign, leg length discrepancy). Range of abduction of the affected hip and information on PT are mandatory to register at all registration time points. Change of abduction was calculated by subtracting the range of abduction at diagnosis from that at the follow-up. If the abduction difference is negative, there is a concomitant decrease in the range of abduction. For this calculation, bilateral affected hips were excluded (15 patients, where 7 had both hips registered in the SPOQ and had LCPD diagnosed in the first hip before the register was established).

### Statistics

Continuous variables were analyzed with a dependent t-test for parametric data. Wilcoxon signed-rank test was used for nonparametric data. When comparing the range of motion across lateral pillar classes the categorical outcome was analyzed with the Kruskal-Wallis test and the Bonferroni correction for multiple tests was applied to adjust significance levels. The Dunn test was performed as a post-hoc test. Linear regression analyses were computed for the prediction model for the range of abduction at follow-up and adjusted for range of motion at diagnosis. All statistical analyses were performed using R statistic software (Version 3.3.3; R Foundation for Statistical Computing, Vienna, Austria), including the “rms”, “magrittr”,” survival”, “car”, “FSA” “ggplot2” and “Gmisc” packages. This study was approved by the Ethics Research Committee in Uppsala, Sweden (registration number 2018/165, date of issue 6 May 2018).

### Study population

**Fig. 1:**
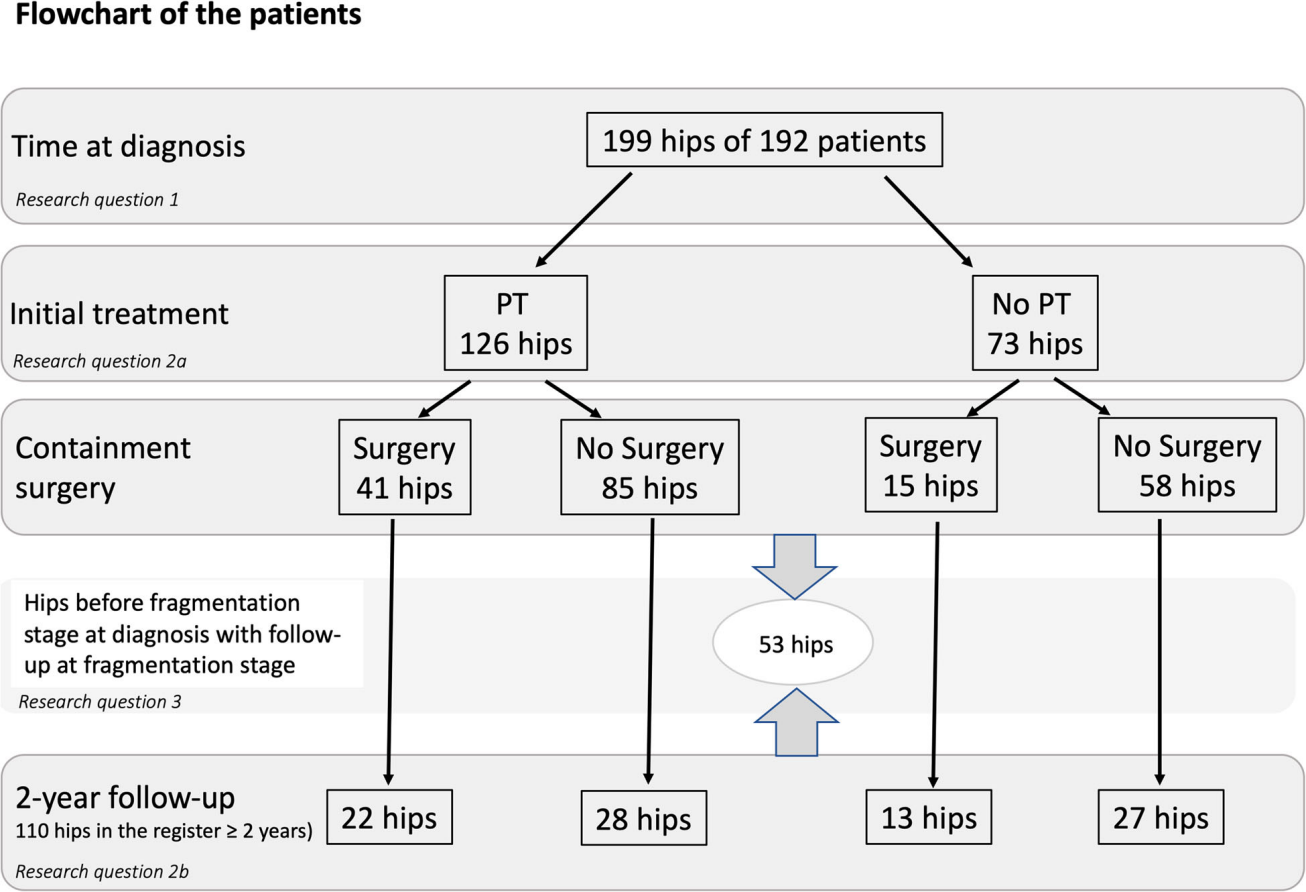
Flowchart of the patients

From the register’s establishment on 1 January 2015 until 31 December 2019, the database contained 199 hips (from 192 patients). We followed the study population from 1 January 2015 until the registration at the 2-year-follow-up before 1 January 2020. The study population’s flowchart is demonstrated in Fig. 1. To evaluate abduction at the time of diagnosis and the given treatment to increase the range of abduction (research questions 1 and 2) we analyzed all 199 hips in the database. To answer the third research question (3) we only included hips that had been too early to classify according to LP at diagnosis and had LP registered either at surgery or the 2-year follow-up (53 hips). The characteristics of the study population are shown in Table 1. Registration of the range of abduction and information on PT is mandatory at all time points.

**Table 1:** Characteristics of the study population

	Level	Overall
n		199
Age at LCPD diagnosis (mean (SD))		5.96 (2.23)
LCPD_Side (%)	right	95 (47.7)
	left	104 (52.3)
Sex (%)	male	156 (78.4)
	female	43 (21.6)
Bilateral LCPD (%)	no	176 (91.2)
	yes	17 (8.8)
Lateral pillar at diagnosis (%)	B	58 (29.1)
	B/C	20 (10.1)
	C	13 (6.5)
	Too early to classify	108 (54.3)

## Results

**Fig. 2:**
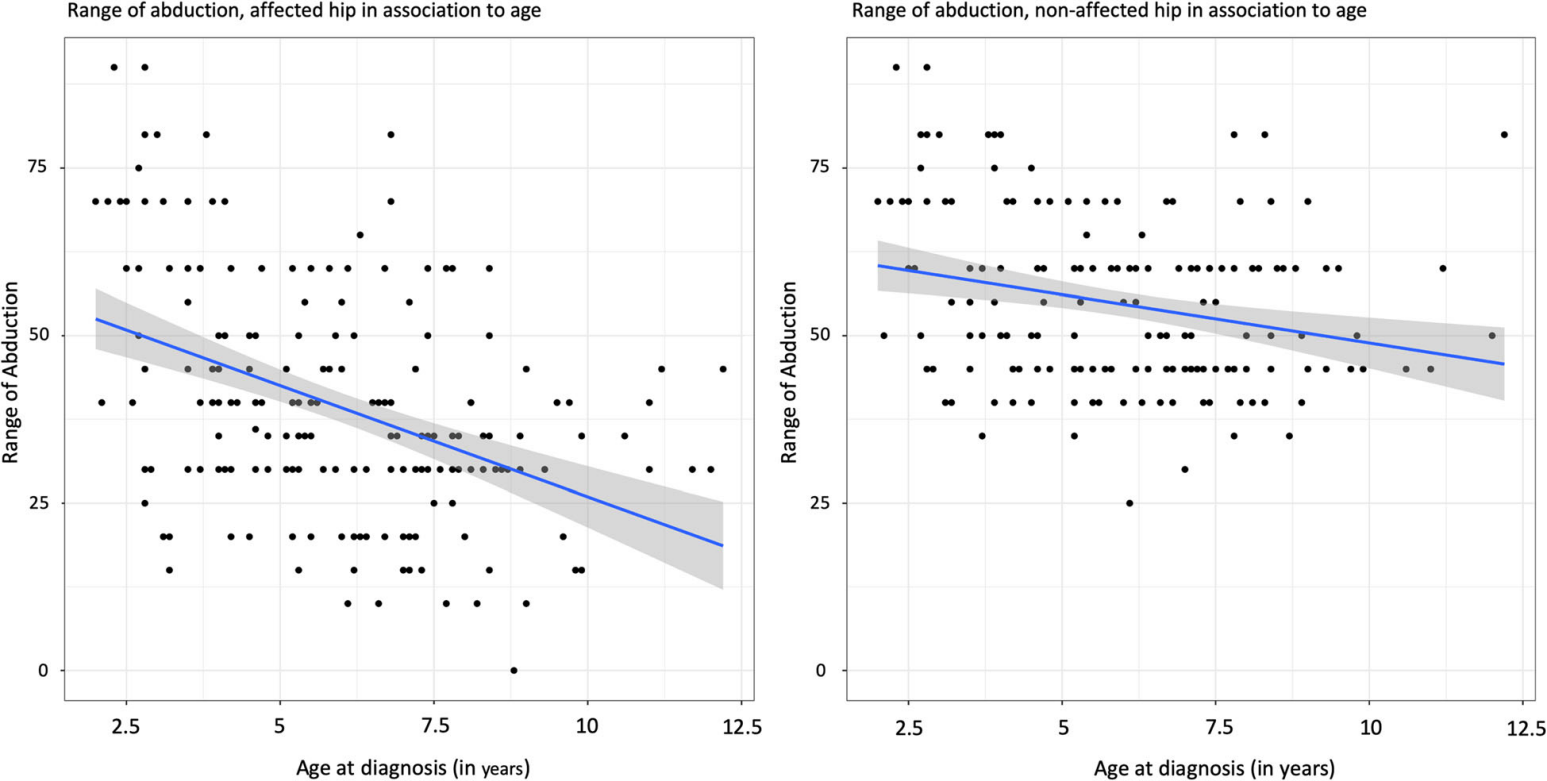
Range of Abduction affected hip in associations to age.

**Fig. 3:**
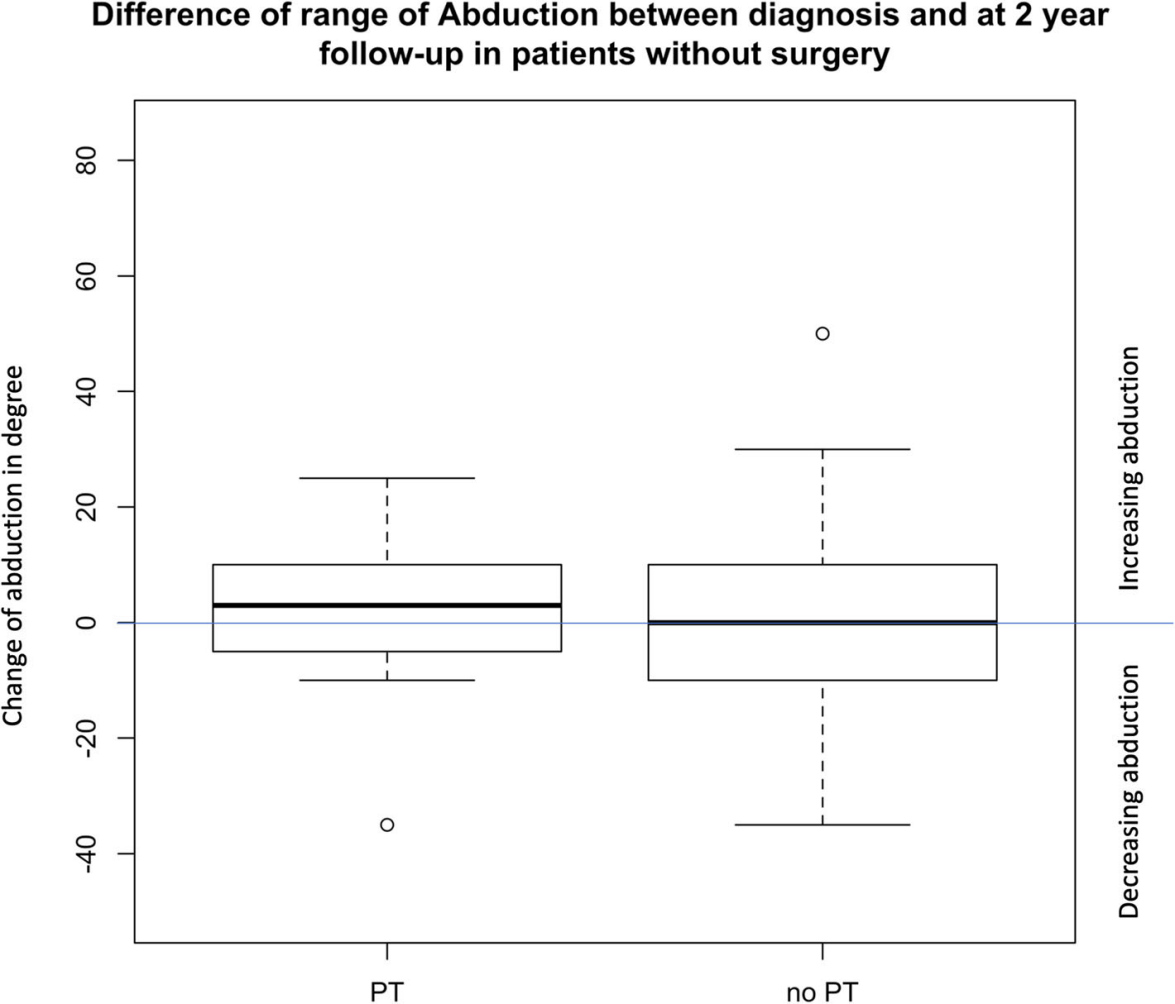
Difference of range of Abduction between diagnosis and at 2 year follow-up in patients without surgery

The mean range of abduction at diagnosis was 39 degrees (range 0 to 90). This mean range was significantly lower compared with the nonaffected hip (54 degrees, range 25 to 90). There were no differences between boys and girls in the abduction of the affected hip at diagnosis or the 2-year follow-up. Younger age was associated with a better range of abduction at diagnosis and the 2-year follow-up (Fig. 2 and 3), with the association more pronounced in the affected hip than in the nonaffected hip (Table 2). A loss of abduction with the passage of time was not associated with age (Estimate [Est]: 0.4, 95% confidence interval [CI]: − 1.4 to 2.3).

**Table 2:** Linear regression analyses to identify the relationship of abduction at diagnosis and follow-up of the affected and nonaffected hip to age at diagnosis (adjusted for sex and abduction at diagnosis). With increasing age (in years) a decrease of range of abduction was seen (Estimate in degree)

Age at diagnosis	Est.	2.5%	97.5%	p
In relation to range of abduction LCPD hip at diagnosis	−3.3	−4.3	− 2.3	< 0.001
In relation to range of abduction nonaffected hip at diagnosis	−1.4	−2.3	−0.6	< 0.001
In relation to range of abduction LCPD hip at follow-up	−3.1	−4.4	−1.8	< 0.001
In relation to range of Abduction nonaffected hip at follow-up	−1.3	−2.5	−0,2	< 0.05

In all, 126 patients (63%) either received instructions for abduction training or were referred to a physiotherapist and 2 patients were additionally treated with abduction brace. Patients with normal range of abduction at baseline had PT prescribed less often than patients with inferior range of abduction. Age or sex had no impact on the prescription practice.

**Fig. 4:**
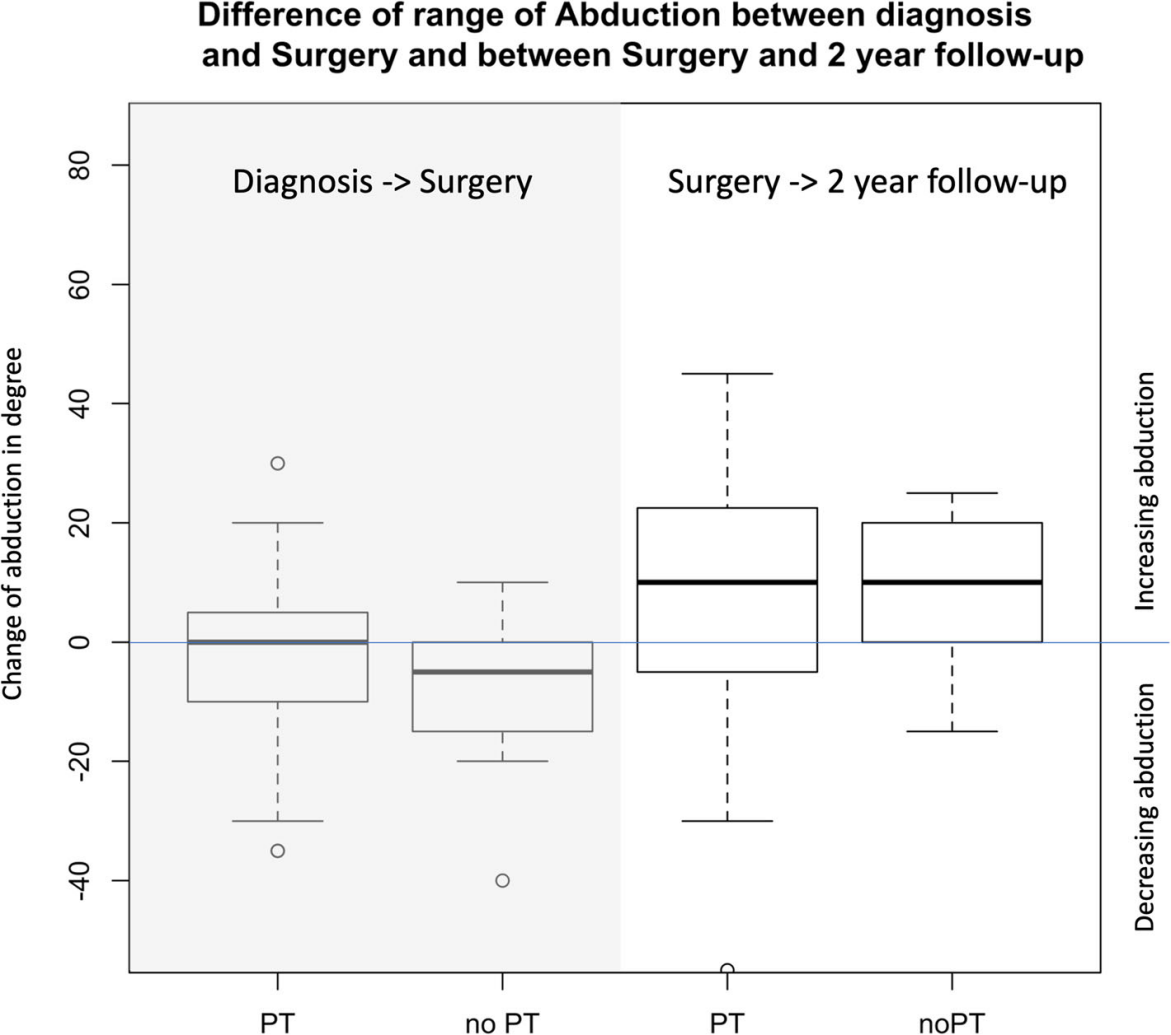
Difference of range of Abduction between diagnosis and Surgery and between Surgery and 2 year follow-up

There was a small trend that the loss of abduction was less in children who received PT than in children who did not receive PT, even when adjusting for abduction at diagnosis (Est: − 0,7, 95% CI: − 2.3-0.8) (Fig. 3). In children who received surgery this trend was seen between time at diagnosis and time of surgery. But not between time of surgery and follow-up (Fig. 4). We had no data on whether patients received PT after surgery.

**Fig. 5:**
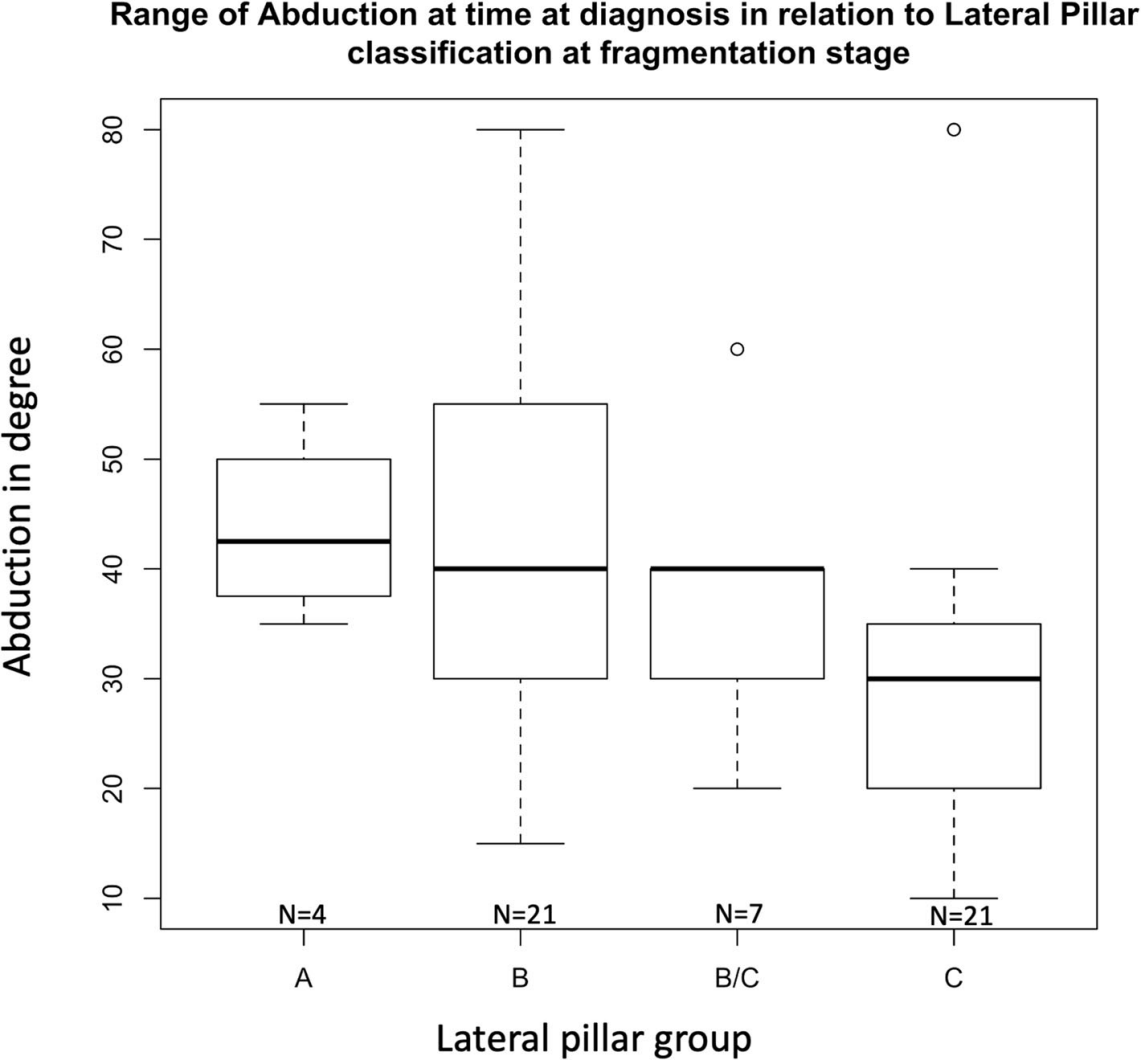
Range of Abduction at time at diagnosis in relation to Lateral Pillar classification of fragmentation stage

Range of abduction at diagnosis in children with hips that are before fragmentation stage correlated significantly with a higher degree of lateral pillar involvement as measured with the lateral pillar classification (Est: − 5.3, 95%, CI: − 10.0 to − 1.1) (Fig. 5).

## Discussion

Range of hip abduction decreased with older age at diagnosis of LCPD and the 2-year follow-up. However, this association was more pronounced in the affected than in the nonaffected hip. Sankar et al. found an age-dependent range of motion of the hip, even in healthy children [14]. In our study no difference in the range of abduction was found between boys and girls, which is in line with the findings of Sankar et al. [14], who observed less range of abduction in boys than in girls only in the older age groups (11–17 years). Almost two thirds of the patients registered in the SPOQ received either instruction for abduction training or were referred to a physiotherapist at diagnosis. Only two patients were treated with an abduction brace.

Brech et al. showed a significant improvement in range of motion in children treated with physiotherapy [2]. According to our results, physiotherapy seems to help to maintain a broader range of abduction in the hip joint, which is favorable for functional outcomes [2]. Maintaining the morphology of the hip joint is the main objective with the treatment to prevent early degeneration and loss of range of motion in young adulthood [10]. The effectiveness of surgical treatment options to maintain the hip’s morphology has been investigated and proven in two prospective multicenter studies [5, 18]. But, controversies exist as to whether surgery is needed in younger children between 6 and 8 years of age at diagnosis [5, 8, 18]. Nevertheless, there is a consensus that a good range of abduction before surgery is vital [3].

Although widely used, a major limitation of the lateral pillar classification is that it can only be applied in the fragmentation stage [9]. We therefore asked if an early decreased range of abduction could be predictive for later lateral pillar classification when the fragmentation stage is reached. We found that patients with a lower range of abduction at the time of diagnosis (before the fragmentation stage) developed a higher degree of lateral pillar involvement as measured with the lateral pillar classification.

### Limitations of the study

This study is a national register study and many orthopedic surgeons register their findings to the SPOQ. We therefore were unable to validate the measurements for abduction. However, instructions, how to measure abduction in a standardized technique are given in the SPOQ. In addition, Sankar et al. found excellent intra- and interobserver agreement in abduction in a study of 504 hips examined by two observers [14]. An expert team of SPOQ which consists of the senior author and 2 additional pediatric orthopedic surgeons with special interest in LCPD validates the radiographic measurement annually by retrieving the radiographs of the different hospitals. Within this validation process the registration of lateral pillar classification had been verified or corrected in 141 hips at time at diagnosis and in 57 hips at follow-up. The agreement between the measurements in SPOQ and measurements of the expert team at time at diagnosis were inferior (ICC 0.74) than at time at follow-up (ICC 0.81). The inferior ICC at time at diagnosis resulted mostly due to the fact that the fragmentation stage at diagnosis was not reached yet but the registrars already classified. Instructions for PT differ from institution to institution according to local traditions and physiotherapists. Ten patients were referred to a physiotherapist and 14 patients only received written instructions for abduction training from the orthopedic department without a referral to the physiotherapist. The vast majority received both (102 patients). The study population was not large enough to analyze different types of PT in a meaningful way. Another drawback was that we were unable to verify the patient’s compliance to follow PT instructions. It is common practice that patients received PT after undergoing surgery; however, PT prescription after surgery is not part of the SPOQ registration and therefore cannot be assured.

None of the patients have undergone an 18-year follow-up examination, which, in the future, will provide more information about the long-term effects of PT.

## Conclusion

The age-dependent decrease of range of abduction is significantly more pronounced in the affected than in the nonaffected hip. There was a trend that PT either maintained or even increased the range of abduction in patients with LCPD. The range of abduction at diagnosis before the fragmentation stage seems to be associated with the involvement of the lateral pillar measured with lateral pillar classification in the fragmentation stage. It seems that PT is beneficial to maintain the range of abduction in patients with LCPD. Further studies are needed to identify which type of PT gives the best results to maintain or increase abduction and should be recommended to patients with LCPD.

## Data Availability

The dataset that is necessary to replicate main findings can be obtained from the author upon reasonable request.

## Abbreviations

CI: Confidence Interval
Est: Estimate
ICC: Intraclass Correlation
LCPD: Legg-Calvé-Perthes diseases
PT: Physiotherapy
SPOQ: Swedish Pediatric Orthopedic Quality
